# The Incidence of IgG4-Related and Inflammatory Abdominal Aortic Aneurysm Is Rare in a 101 Patient Cohort

**DOI:** 10.3390/jcm12124029

**Published:** 2023-06-13

**Authors:** Maja Carina Nackenhorst, Marvin Kapalla, Simon Weidle, Felix Kirchhoff, David Zschäpitz, Sabine Sieber, Christian Reeps, Hans-Henning Eckstein, Heike Schneider, Markus Thaler, Philipp Moog, Albert Busch, Nadja Sachs

**Affiliations:** 1 Department of Pathology, Medical University of Vienna, 1090 Vienna, Austria; 2 Division of Vascular and Endovascular Surgery, Department for Visceral, Thoracic and Vascular Surgery, Medical Faculty Carl Gustav Carus and University Hospital, Technische Universität Dresden, 01307 Dresden, Germany; 3 Department for Vascular and Endovascular Surgery, Klinikum rechts der Isar, Technical University Munich, 80333 Munich, Germany; 4 Department of Surgery, Klinikum rechts der Isar, School of Medicine, Technical University Munich, 80333 Munich, Germany; 5 German Center for Cardiovascular Research (DZHK), Partner Site Munich Heart Alliance, 10785 Berlin, Germany; 6 Institute of Clinical Chemistry and Pathobiochemistry, Klinikum rechts der Isar, Technical University Munich, 80333 Munich, Germany; 7 Department of Nephrology, School of Medicine, Technical University Munich, 80333 Munich, Germany

**Keywords:** abdominal aortic aneurysm, immunoglobulin G4, IgG4-related disease, inflammatory aneurysm

## Abstract

Abdominal aortic aneurysms (AAA) are the most frequent aortic dilation, with considerable morbidity and mortality. Inflammatory (infl) and IgG4-positive AAAs represent specific subtypes of unclear incidence and clinical significance. Here, histologic and serologic analyses with retrospective clinical data acquisition are investigated via detailed histology, including morphologic (HE, EvG: inflammatory subtype, angiogenesis, and fibrosis) and immunhistochemic analyses (IgG and IgG4). In addition, complement factors C3/C4 and immunoglobulins IgG, IgG2, IgG4 and IgE were measured in serum samples and clinical data uses patients’ metrics, as well as through semi-automated morphometric analysis (diameter, volume, angulation and vessel tortuosity). A total of 101 eligible patients showed five (5%) IgG4 positive (all scored 1) and seven (7%) inflammatory AAAs. An increased degree of inflammation was seen in IgG4 positive and inflAAA, respectively. However, serologic analysis revealed no increased levels of IgG or IgG4. The operative procedure time was not different for those cases and the short-term clinical outcomes were equal for the entire AAA cohort. Overall, the incidence of inflammatory and IgG4-positive AAA samples seems very low based on histologic and serum analyses. Both entities must be considered distinct disease phenotypes. Short-term operative outcomes were not different for both sub-cohorts.

## 1. Introduction

Abdominal aortic aneurysms (AAA) are the most frequent aortic aneurysm and have an age- and sex-dependent prevalence of 1.5–8% in the elderly male population [1]. Its most feared complication is rupture, with still high morbidity and mortality rates, even in modern vascular practice, where endovascular aortic repair (EVAR) has outnumbered traditional open repair (OAR). Hence, international guidelines recommend elective repair, based on a diameter threshold, where rupture risk outweighs perioperative mortality [2,3].

The initial cause of AAA still remains a matter of debate, as well as why a specific patient develops an aneurysm. Apart from the occasional presence within heritable connective tissue disorders, no single genetic trait is currently held responsible for aortic dilation. However, the most common features observed in tissue specimens assembled during OAR pinpoint to a proteolytic imbalance with vast destruction of the elastic lamellae in the tunica media harboring extensive tissue remodeling with a thickened vessel wall, calcifications, angiogenesis, and vascular smooth muscle cell phenotype switch [1,4,5]. Secondly, an abundant intraluminal thrombus is considered as an enzymatically active layered visco-elastic element contributing to vessel wall degradation [1,6]. Additionally, the immune system might be critically involved in either promoting or inhibiting aortic wall dilation, since both humoral and cellular-mediated pro- as well as anti-inflammatory responses have been extensively reported [5,7,8,9].

In a minority of patients, approx. 5–10%, so-called inflammatory AAA (inflAAA) has been described as a possible link to autoimmune diseases such as giant cell arteritis or Takayasu’s disease with a thickened aortic wall (so-called mantle sign) and possible retroperitoneal fibrosis [10,11]. Here, the involvement of the recognized systemic inflammatory disorder known as immunoglobulin G4 (IgG4)-related disease has been linked to AAA by many groups, suggesting approx. 5–10% of all AAAs; however, up to 57% of inflAAA cohorts appear to be IgG4 positive to varying degrees [12,13,14,15,16]. Hence, diagnostic criteria involving both histology and blood sampling have been established to define inflammatory and IgG4-positive AAA. Uncertainty remains about the true prevalence of both entities and the ideal treatment strategy [15,17].

Hence, in this study, we aimed to determine the percentage of inflammatory and IgG4-related AAA upon serologic and histopathologic analyses in a single center cohort and investigated possible implications of IgG4-positive cases on short-term clinical outcomes.

## 2. Patients and Methods

### 2.1. Patient Identification, Inclusion Criteria and Ethical Approval

Patients with AAA were identified retrospectively from a large aortic database in connection to a biobank, as described previously, from 2007–2019 [18,19]. Clinical data was retrieved from electronic patient records (see below).

Inclusion criteria were a full thickness sample from the left anterior wall of the AAA sac during OAR, enabling detailed histologic analysis with successful IgG4 staining (see below) and a valid serum sample. Additionally, corresponding clinical and patient data (see below) had to be available. Indications for open repair were surgical reasons, patient will, or operator’s choice, in line with international guidelines [2,3].

Patient data were pseudonymized for biobanking and anonymized for further analysis. The study was performed in accordance with the declaration of Helsinki and tissue sampling was approved by the local ethics committee (Ethikkommission Klinikum rechts der Isar: 2799/10). The specific study was approved additionally (Ethikkommission Klinikum rechts der Isar: 576/18S).

### 2.2. Basic Patient and Clinical Data, Procedure Details and Outcomes

Basic clinical data included age, sex (male/female), AAA state (symptomatic, ruptured, asymptomatic), maximum diameter (maximum transverse diameter applying multiplane reconstructions from 1–5 mm, CT-angiographies right before OAR, measurements performed by experienced vascular surgeon), type of AAA (infra-, juxta-, suprarenal; infrarenal = neck length ≥ 10 mm), concomitant iliac aneurysm (one/two sided; common iliac artery ≥ 25 mm), co-morbidities (hypertension, diabetes, hyperlipidemia, coronary artery disease CAD, chronic obstructive pulmonary disease COPD, peripheral artery disease PAD), smoking (current/ex/never), medication (anti-thrombocyte-aggregation, angiotensin-converting-enzyme inhibitor, statin, metformin/insulin), and laboratory results (C-reactive protein CRP, leukocyte/thrombocyte count, serum creatinine).

Body surface area was calculated by the formula of DuBois and aortic size index (ASI), accordingly [20,21]. Psoas volume and area were calculated using the Brainlab Buzz© and the included Elements SmartBrush© (both Brainlab, Munich, Germany) application for semi-automated psoas volume and area calculation.

Procedural data included the operation time, the type of reconstruction (tube vs. bifurcated Y graft), and the access (trans- vs. retroperitoneal). Any additional anastomosis (i.e., re-insertion of the inferior mesenteric artery or renal bypass) were summarized. 

For short-term clinical outcomes, the days in hospital and in the intensive care unit (ICU) were counted. In-hospital complications included surgical (surgical site infection (SSI), leg ischemia, bleeding, bowel ischemia) and medical (urinary tract infection, acute kidney failure, ischemic colitis, myocardial infarction, stroke, lung edema, pneumonia) events. Additionally, the in-hospital mortality was calculated.

### 2.3. AAA Characteristics

The morphologic analysis (diameter, volume, ratios, angulation, calcification, and vessel tortuosity indices) was performed semi-automatically with Endosize© (Therenva, Rennes, France), a software for clinical assessment of AAAs as well as for EVAR planning, as previously described [19].

The state and extent of AAA were identified from the electronic patient file. Additionally, all preoperative CT angiographies were reviewed by two vascular surgeons for signs of rupture, extent of AAA, and mantle signs suggestive of inflammatory AAA [11].

### 2.4. Sample Acquisition, Preparation and Digitalization

After removal from the intraoperative situs, tissue was immediately transferred to a chilled phosphate-buffered saline for transport and further processing in the laboratory. 

Samples were fixed in formalin (4% PFA) for 24 h and if necessary, decalcification on an EDTA basis (Entkalker soft SOLVAGREEN^®^, Carl ROTH, Karlsruhe, Germany) was performed for 2–7 days. Subsequently, specimens were prepared for paraffin embedding in standard size (40 × 28 × 6.8 mm) POM histology cassettes (Kartell, Noviglio, Italy). Sections (2 µm) of paraffin-embedded samples were mounted on glass slides (Menzel SuperFrost, 76 × 26 × 1 mm, Fisher Scientific, Schwerte, Germany).

Afterwards, Hematoxylin-eosin (HE) (ethanolic eosin Y solution, Mayer’s acidic hemalum solution, Waldeck, Münster, Germany) as well as Elastica van Gieson (EvG) (picrofuchsin solution after Romeis 16th edition, Weigert’s solution I after Romeis 15th edition) stainings were performed according to the manufacturer’s protocol. Slides were covered using Pertex (Histolab products, Askim, Sweden) as the mounting medium and glass coverslips (24 × 50 mm, Engelbrecht, Edermünde, Germany).

Slides (including immunohistochemistry) were then scanned with Aperio AT2 (Leica, Wetzlar, Germany). Scanned slides were analyzed and prepared for composite figures using QuPath-0.3.2 open-source software [22].

### 2.5. Immunohistochemistry

FFPE sections used for immunohistochemistry were mounted on poly-l-lysine (Merck, Darmstadt, Germany) pretreated glass slides (SuperFrost PLUS, Epredia Europe, Basel, Suisse). The sections were incubated for a minimum of 48 h at 60 °C and afterwards were de-paraffinized. Demasking of the antibody binding sites was achieved by cooking the slides for 7 min in citrate acid (pH 6). After every following step the samples were rinsed in Tris-buffer (Trizma base, NaCl, Merck, Darmstadt, Germany). Endogenous peroxidase activity was quenched by incubating the slides for 15 min with 3% hydrogen peroxide (Merck, Darmstadt, Germany). Subsequently, the sections were incubated with the respective primary antibody (IgG: EPR4421, abcam, 1:10,000; IgG4: EP4420, abcam, 1:3000). Dako REAL Antibody Diluent (Dako, Glosirup, Denmark) was used for antibody dilution. Target staining was done by incubating the samples for 25 min with the biotinylated secondary antibody, followed by incubation for 25 min, adding streptavidin peroxidase, and additional incubation for 2–3 min with DAB+ chromogen, diluted in horseradish peroxidase substrate buffer (Dako REAL Detection System Peroxidase/DAB+, Rabbit/Mouse Kit; Dako, Glosirup, Denmark). Counterstaining was done with Mayer’s hemalum solution (Carl Roth, Karlsruhe, Germany). The sections were dehydrated and subsequently covered, as described above. Antibody specificity was tested and evaluated on tonsil sections. Here, control incubations were performed with the secondary antibody only.

### 2.6. Pathologic Analysis and Definitions

All samples were investigated by a pathologist (MCN). Detailed analysis included:

Intima: The American Heart Association (AHA) classification for atherosclerotic lesions was applied to the intima where possible [23].

Media: The inflammation in the media was scored according to the degree of inflammation (0 = no or only singular inflammatory cells, 1 = low degree of inflammation in form of singular small infiltrates, 2 = intermediate degree of inflammation in form of localized and diffuse infiltrates, and 3 = high degree of inflammation with diffuse dense infiltrates). 

Adventitia: Adventitial features were scored according to degree of inflammation (0 = no inflammation, 1 = low degree of inflammation, 2 = intermediate degree of inflammation, and 3 = high degree of inflammation), composition of inflammatory infiltrate (1 = mainly composed of mononuclear cells, 2 = granulocytes, 3 = plasma cells, or 4 = mixed infiltrate), and degree of fibrosis (0 = no fibrosis, 1 = low degree of fibrosis, 2 = intermediate degree of fibrosis, and 3 = high degree of fibrosis). 

Immunohistochemical staining for IgG and IgG4 was scored as follows, according to established definitions [14,24]:

0 = no positive cells detectable;

1 = singular positive cells (≤20 cells/HPF);

2 = few positive cells and small clusters (≤50 cells/HPF);

3 = abundance of positive cells (≥50 cells/HPF).

### 2.7. Serum and Blood Analysis and Definitions

Complete blood counts were performed on a XE-5000 or XN-10 device from Sysmex (Norderstedt, Germany) in order to determine leukocyte and thrombocyte counts. Patient serum was obtained using Serum-Gel S-Monovettes (Sarstedt, Nümbrecht, Germany), aliquoted, and stored at −80 °C until further analysis. C-reactive protein was measured on the c702 turbidimetry module of the cobas 8000 analyzer from Roche (Mannheim, Germany). Complement factors C3 and C4 were also measured with turbidimetric assays on the c502 module as well on the cobas 8000 device. IgG, IgG2, IgG4, and IgE were quantified on the BN Prospec nephelometer from Siemens (Eschborn, Germany).

### 2.8. Statistics

Statistical analysis was performed using IBM SPSS for Windows, Version 26.0 (IBM Corp., Armonk, NY, USA). All clinical characteristics were grouped to build categorical or nominal variables. Dichotomous variables were recorded as absolute frequencies (number of cases) and relative frequencies (percentages). Continuous data are presented as mean and standard deviation or median and interquartile range (IQR).

## 3. Results

Overall, 101 patients (87% male, 67 ± 8.2 years old) met the inclusion criteria, having both histologic and serologic samples available for analysis (Supplementary Figure S3). The patients’ baseline characteristics are shown in Table 1. The majority of cases were asymptomatic (83%) infrarenal (50%) AAAs with a mean diameter of 58 ± 11.1 mm.

**Figure 1:**
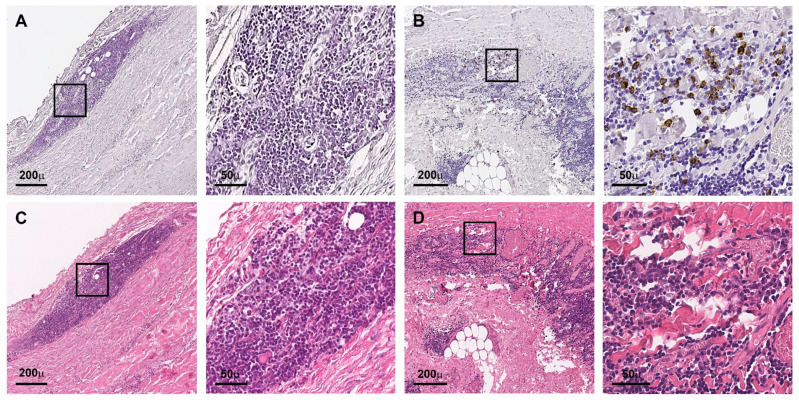
IgG4 positive AAA staining: (**A**,**B**) overview and close up of IgG4-immunostaining (negative in (**A**), positive in (**B**) from two distinct patients. (**C**,**D**) Corresponding overview and close up of HE staining (scale bar 200 µm, 50 µm, respectively, all photos lumen oriented upwards).

**Figure 2:**
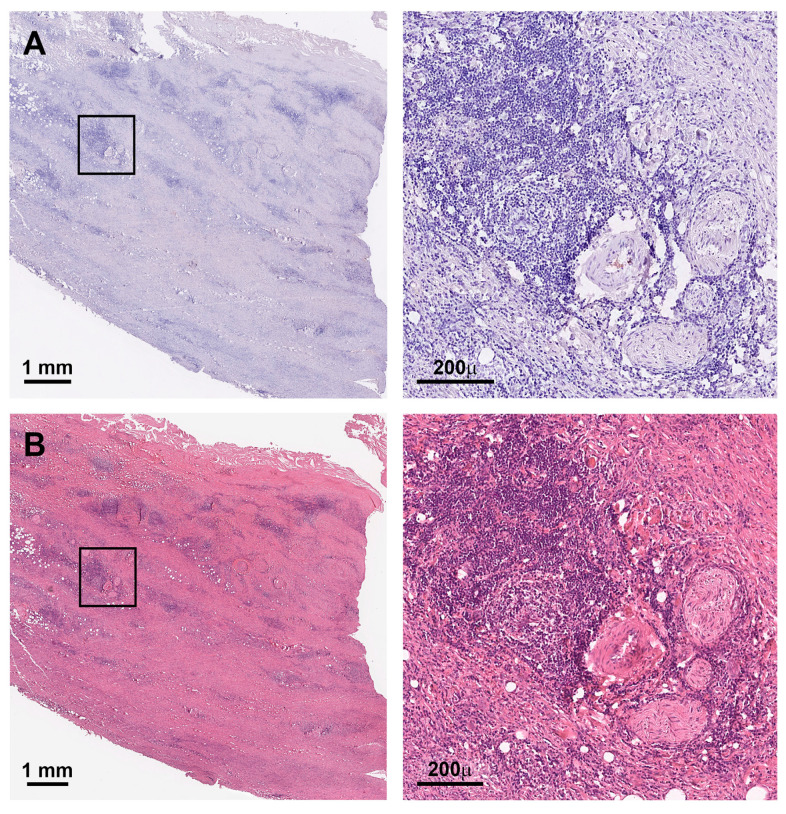
Inflammatory AAA histomorphology. (**A**) Inflammatory AAA with no IgG4 positivity in overview and close up. (**B**) Corresponding HE staining with high degree of wall thickening with layered inflammatory infiltrates in the entire vessel wall (scale bar 200 µm, 1 mm respectively; all photos lumen oriented upwards).

In this cohort, seven samples were considered inflammatory AAAs (7%) and another five samples were considered IgG4 positive (5%) based on histologic characterization (Figure 1 and Figure 2). No inflAAA was found to be IgG4 positive or vice versa. Based on a scale of 0–3 for the amount of IgG4 positivity defined by Raparia et al., all five positive samples in this study reached score 1, respectively (Figure 1B,D, Supplementary Figure S1) [14]. However, patients with inflammatory AAAs had a lower BMI and average psoas volume, including a higher percentage of females in this small sub-cohort (Table 1). Sufficient aneurysm growth data were only available for 48% of patients and could not reliably be analyzed (Supplementary Table S1).

**Figure 3:**
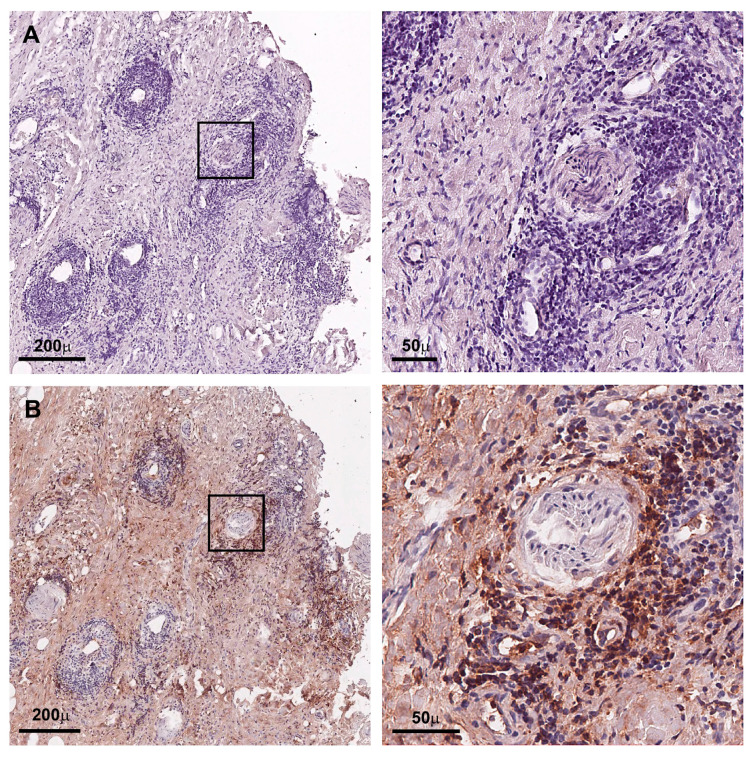
IgG and corresponding IgG4 positivity. (**A**) AAA tissue sample with IgG4 immunohistochemistry with lack of positive cells and corresponding high level of IgG-positive cells (**B**) (scale bar 200 µm, 50 µm, respectively).

A detailed histologic comparison revealed an elevated adventitial degree of inflammation in the IgG4-positive samples (1.3 ± 0.7 vs. 1.8 ± 0.4) and the inflAAA samples (1.3 ± 0.7 vs. 2.2 ± 0.9), respectively (Table 2, Supplementary Figure S2). No differences were seen regarding the predominant type of inflammatory cells. A high number of IgG-positive cells did not correspond to IgG4 positivity (Figure 3). Interestingly, IgG and especially IgG4 serum levels were not altered in the histologically positive patients (Table 2). Detailed serologic analysis only showed a significantly elevated thrombocyte count in patients with inflAAA (215 ± 55.9 × 10^3^/µL vs. 267 ± 49.6 × 10^3^/µL). Notably, none of these seven patients showed a distinct mantle sign on the pre-operative CT angiography. 

Finally, no significant differences in OAR procedure time were seen for these two sub-cohorts and also the immediate outcomes length of stay, medical/surgical complication rate, and in-hospital mortality did not differ (Table 3, Supplementary Table S1).

## 4. Discussion

This study shows a very low incidence of IgG4 positivity and comparable incidence of inflammatory AAA samples based on a detailed histologic analysis in a central European single center patient cohort. Additionally, the vast heterogeneity between histologic and serologic IgG4 diagnostic criteria and findings is emphasized. However, clinical appearance, AAA anatomy, and short-term operative outcomes do not differ between these two sub-cohorts and the overall patient set, and serum analysis did not show distinct levels of immunoglobulins. 

Awareness of IgG4-related diseases, first described for autoimmune pancreatitis, has dramatically increased over the last two decades [25]. Here, patients initially operated on for suspected malignancy upon imaging are nowadays treated with steroids. Ever since, the variety of IgG4-related disease classifications has been expanded to every other organ system. Accordingly, classification schemes based on histologic features have been put forward, with the number of IgG4-positive cells being a distinct hallmark [24,26]. As for AAA, reports on a possible autoimmune manifestation date back to the 1980s, however, the first description of IgG4-positive AAA cohorts stem from two independent Japanese groups in 2008 [16,27].

In these studies, inflAAA was suspected to be rather more IgG4 positive than typical atherosclerotic specimens and IgG4-positive cell infiltration was reported in 50–100% of inflammatory samples [16,28].

In our study, none of the inflammatory cases showed IgG4 positivity (Figure 2). Despite this high IgG4 burden in studies from Japan, Koo et al. reported 3 out of 29 positive aortic cases (10.9%) from Korea [29]. The largest study to date stems from the Czech Republic and reported 7 out of 114 AAA patient samples (6.1%) that were positive for IgG4-related disease, comparable to the results shown here (Table 1, Figure 1, Supplementary Figure S1) [13]. Hence, one could hypothesize a different disease distribution over different ethnicities; this may, however, harbor selection and reporting bias, also including aneurysm sizes and emergency procedures, when, i.e., no sample can be acquired during surgery. Despite the significantly higher adventitial degree of inflammation seen in our cohort, the IgG4 positivity score (= # of positive cells per high power field) was one in all samples (Figure 1, Supplementary Figure S1, Table 2) [14,16]. Interestingly, no change in the distribution of type of inflammatory infiltrate was found (Table 2). Regarding inflAAA, reports are more frequent; thus, the overall prevalence of approx. 5% among all patients might better reflect reality [11]. To account for that, we also applied a strict histologic criteria for inflAAA (Figure 2) [14]. Although very clear upon histologic analysis, we did not see a typical mantle sign, suspected in 70–100% of cases, in the seven patients deemed positive in this study. However, an abundant variance in aneurysm wall thickness, even in inflAAA, has been reported before and might affect the appearance on CT [4,30,31].

Later, the specter of IgG4-related disease was expanded to the thoracic aorta, the aortic arch, and the pericardium [32,33,34]. However, in all reported cohorts, the IgG4 serum levels only partially reflected histologic positivity [24,35]. In our study, none of the IgG4-positive patients had significantly increased IgG or IgG4 serum levels (Table 2). Hence, the question remains on how to best diagnose the disease, since biopsies from AAAs are not feasible and serum immunoglobulin levels are not adequate [24,36]. Generally, the incidence of IgG4 positivity in non-aneurysmatic (peri-)aortitis seems to be rather low [37]. Yet, in patients with diagnosed IgG4-related disease, aortic dilation might be a frequent feature [12,38].

Regarding treatment of IgG4-related AAA and inflammatory AAA, studies have reported the equal effectiveness of EVAR and OAR [12,15]. In our study, all patients had OAR with no significant differences in procedural characteristics or short-term clinical outcomes (Table 3). Notably, similar AAA morphologic features were observed for the entire patient cohort as well as inflAAA and IgG4-related disease (Table 1). Interestingly, steroid treatment for IgG4-positive cardiovascular lesions has been reported alone or in combination with surgical treatment to reduce perioperative complications [39,40]. However, the weight of steroids in the treatment of AAA disease remains unclear and successful surgical repair without steroids has been reported and is shown here (Table 3). 

The study is, of course, limited by the relatively small number of patients, given the incidences of both inflAAA and IgG4-positive AAA (Table 1). Thus, any statistical analysis other than descriptive is not justified, since no conclusion could be drawn. Given the heterogeneity of reports on the subject as presented above, we wanted to ensure histologic and corresponding serologic samples from the same patient [24,28,34]. Naturally, the sample analyzed is only a small cut-out from the entire aneurysm, typically from the left anterior wall during OAR. Thus, histologic appearance might be different at other points of the circumference. This limitation occurs in every histologic study of this kind, yet might harbor severe observational bias. Given these circumstances, conclusions can only be drawn very carefully and warrant further validation in larger patient cohorts.

## 5. Conclusions

In a single-center German AAA patient cohort, the incidence of inflammatory and IgG4 positive samples might be very low and both entities have to be considered distinct disease phenotypes. Diagnosis seems most valid after postoperative histologic analysis. In this cohort, short-term operative outcomes were similar for both sub-groups compared to standard AAA open repair. 

## Figures and Tables

**Table 1:** Patient and AAA characteristics. m = male; f = female; - = not known; x = yes; BMI = body mass index; body surface area is calculated after DuBois; CAD = coronary artery disease; COPD = chronic obstructive pulmonary disease; and ACE = angiotensin converting enzyme.

			Patient Cohort	IgG4-Positive AAA					Inflammatory AAA						
			*n* = 101	*n* = 5					*n* = 7						
Patient Characteristics, Co-morbidities and Frailty															
sex (male)			88 (87.1)	m	m	m	f	m	m	f	m	f	f	m	m
metrics	age (y; mean ± SD)		67 ± 8.2	70	63	71	70	62	69	76	65	64	74	71	55
	height (cm)		176.3 ± 7.5	176	187	-	-	-	178	175	173	165	168	189	-
	weight (kg)		83.4 ± 14.1	90	95	-	-	-	101	74	70	70	41	85	-
	BMI		26.7 ± 3.7	29	27	-	-	-	32	24	23	26	15	24	-
	body surface (m^2^)		1.99 ± 0.19	2.05	2.20	-	-	-	2.2	1.9	1.8	1.8	1.4	2.1	-
	aortic size index (cm/m^2^)		2.99 ± 0.68	2.97	2.32	-	-	-	2.6	2.4	3.4	3.0	3.7	2.4	-
	psoas volume (cm^3^)		188.2 ± 57.5	222	256	-	-	-	210	130	190	91	32	174	-
	psoas area (cm^2^)		18.77 ± 5.8	23.3	28.6	-	-	-	26.8	11.6	13.2	5.9	3.6	20.9	-
co-morbidities	hypertension		83 (82.2)	x	x		x	x	x		x	x	x	x	x
	diabetes		13 (12.9)												
	hyperlipidemia		59 (58.4)		x	x	x	x	x		x		x	x	x
	CAD		42 (41.6)		x		x		x			x	x		
	COPD		21 (20.8)				x	x							x
	PAOD		26 (25.7)				x				x		x		
	renal insufficiency		27 (26.7)	x										x	
	dialysis		2 (2)												
	smoking (current/ex)		81 (80.2)		x	x	x	x	x	x	x	x	x		x
medication	platelet inhibitor		64 (63.4)	x	x	x		x	x	x		x	x	x	x
	ACE inhibitor		32 (31.7)		x		x					x			x
	statin		51 (50.5)	x	x		x	x		x		x			x
	metformin		3 (3)												
	insulin		2 (2)												
AAA Characteristics															
diameter (mm)			58 ± 11.1	61	51	55	64	73	56	46	62	53	53	50	56
volume (cm^3^)			186 ± 121	273	132	215	-	316	202	74	257	156	191	117	99
ratio lumen/total volume			0.54 ± 0.19	0.61	0.64	0.63	-	0.49	0.63	0.73	0.23	0.44	0.27	0.48	0.58
extent	infrarenal		50 (49.5)		x		x	x		x					x
	juxtarenal		32 (31.7)	x		x			x		x	x	x	x	
	suprarenal		19 (18.8)												
	+ iliac aneurysm	uni	10 (9.9)								x				
		bi	12 (11.9)					x	x						
state	symptomatic		8 (7.9)				x								
	asymptomatic		84 (83.2)	x	x	x		x	x	x	x	x	x	x	x
	ruptured		9 (8.9)												
Endosize^®^	α angulation (°)		20 ± 20.2	12	14	24	11	50	45	13	23	22	13	13	43
	β angulation (°)		32.6 ± 14.8	36	12	47	39	54	52	53	31	46	17	17	63
	aortic tortuosity index		1.1 ± 0.06	1.2	1.0	1.1	1.2	1.2	1.1	1.2	1.0	1.1	1.1	1.0	1.3
	iliac tortuosity index		1.35 ± 0.22	1.2	1.3	1.3	1.4	1.7	1.5	1.4	1.5	1.1	2.4	1.3	1.3
	iliac calcification (%)		8.6 ± 11.8	7	12	10	2	1	-	3	0	6	7	5	2

**Table 2:** Histology and serum characteristics. x = yes; normal range: leucocytes (4.0–9.0 × 10^3^/µL); thrombocytes (150–450 × 10^3^/µL); C-reactive protein (CRP) (<0.5 mg/dL); sC3 (90–180 mg/dL); sC4 (10–40 mg/dL); IgG (700–1600 mg/dL); IgG2 (150–500 mg/dL); IgG4 (3–200 mg/dL); and IgE (25–100 IU/mL).

				Patient Cohort	IgG4-Positive AAA					Inflammatory AAA						
				*n* = 101	*n* = 5					*n* = 7						
Histopathologic Features																
adventitia	degree inflammation (0–3)			1.3 ± 0.69	1	2	2	2	2	2	3	1	2	3	1	3
	no		type of inflammation	9 (8.9)												
	monocyte			49 (48.5)	x	x	x	x		x	x		x	x	x	
	granulocyte			1 (1)												
	plasma cell			30 (29.7)					x							
	mix			12 (11.9)								x				x
media	degree inflammation (0–3)			0.3 ± 0.5	1	0	1	0	0	0	0	1	0	0	1	0
	no		type of inflammation	68 (67.3)		x		x	x	x	x		x	x		x
	monocyte			24 (23.8)	x		x								x	
	granulocyte			0												
	plasma cell			5 (5)								x				
	mix			4 (4)												
intima	5	AHA classification		11 (10.9)							x	x				
	6			76 (75.2)		x	x	x	x	x			x	x	x	x
	7			0												
	8			12 (11.9)	x											
Serologic Analysis																
leucocyte count (×10^3^/µL)				7.9 ± 2.5	7.8	8.5	5.1	6.1	8.0	8.2	6.8	10.8	11.6	8.8	9.6	7.4
thrombocyte count (×10^3^/µL)				215.2 ± 55.9	284	206	202	229	215	261	336	323	200	279	223	250
C-reactive protein (mg/dL)				1.5 ± 4.5	1.2	0.9	0.9	0.7	0.8	1	0.8	1	0.9	0.5	1.5	0.9
sC3 (mg/dL)				128.4 ± 26.8	127	150	74	220	179	111	145	127	135	114	86	101
sC4 (mg/dL)				25.8 ± 8.3	21	26	13	47	27	20	36	33	36	30	25	17
IgG (mg/dL)				1000 ± 278.6	923	1160	476	500	560	1840	770	874	785	1210	755	657
IgG2 (mg/dL)				319.3 ± 143.3	275	405	135	127	-	642	192	239	177	188	129	250
IgG4 (mg/dL)				92.5 ± 102	27	61	51	24	29	59	6.3	79	64	34	17	38
IgE (IU/mL)				330.1 ± 1004	10	90	4	192	-	21	4	237	17	81	157	41

**Table 3:** Procedural and outcome details. x = yes; Additional complications are shown in Supplementary Table S1.

	Patient Cohort	IgG4-Positive AAA					Inflammatory AAA						
	*n* = 101	*n* = 5					*n* = 7						
Procedural Details													
tube graft (vs. Y graft)	53 (52.5)	x	x	x				x		x	x	x	x
retroperitoneal access	47 (46.5)	x	x					x					x
additional anastomosis (any)	16 (15.8)											x	
procedure time (min)	242.1 ± 94.9	227	176	233	165	145	257	132	320	178	149	181	218
Clinical Outcome													
days in-hospital	15 ± 11	11	7	7	12	9	8	7	13	9	12	8	7
days on ICU	4 ± 5	1		3	3	3	8	7	1	1	2	1	1
surgical complication	20 (19.8)				x				x	x			
medical complication	40 (39.6)	x	x							x			
in-hospital mortality	3 (3.0)												

## Data Availability

All available data for this study has been included in the manuscript or the supplement.

## Supplementary Materials

The following supporting information can be downloaded at: https://www.mdpi.com/article/10.3390/jcm12124029/s1, Figure S1: Additional example of IgG4 positive AAA staining. Figure S2: Degree of inflammation in AAA vessel wall. Figure S3: Inclusion flow chart. Table S1: Additional AAA characteristics and short-term clinical outcomes.
