# Supplementary material for: The Incidence of IgG4-Related and Inflammatory Abdominal Aortic Aneurysm Is Rare in a 101 Patient Cohort
Source: J Clin Med. 2023 Jun 13;12(12):4029. doi: 10.3390/jcm12124029 (PMC10298928; doi:10.3390/jcm12124029)
Supplement: Supplementary file 1 [file jcm-12-04029-s001.zip › jcm-2398779-supplementary.pdf]

Supplement Material

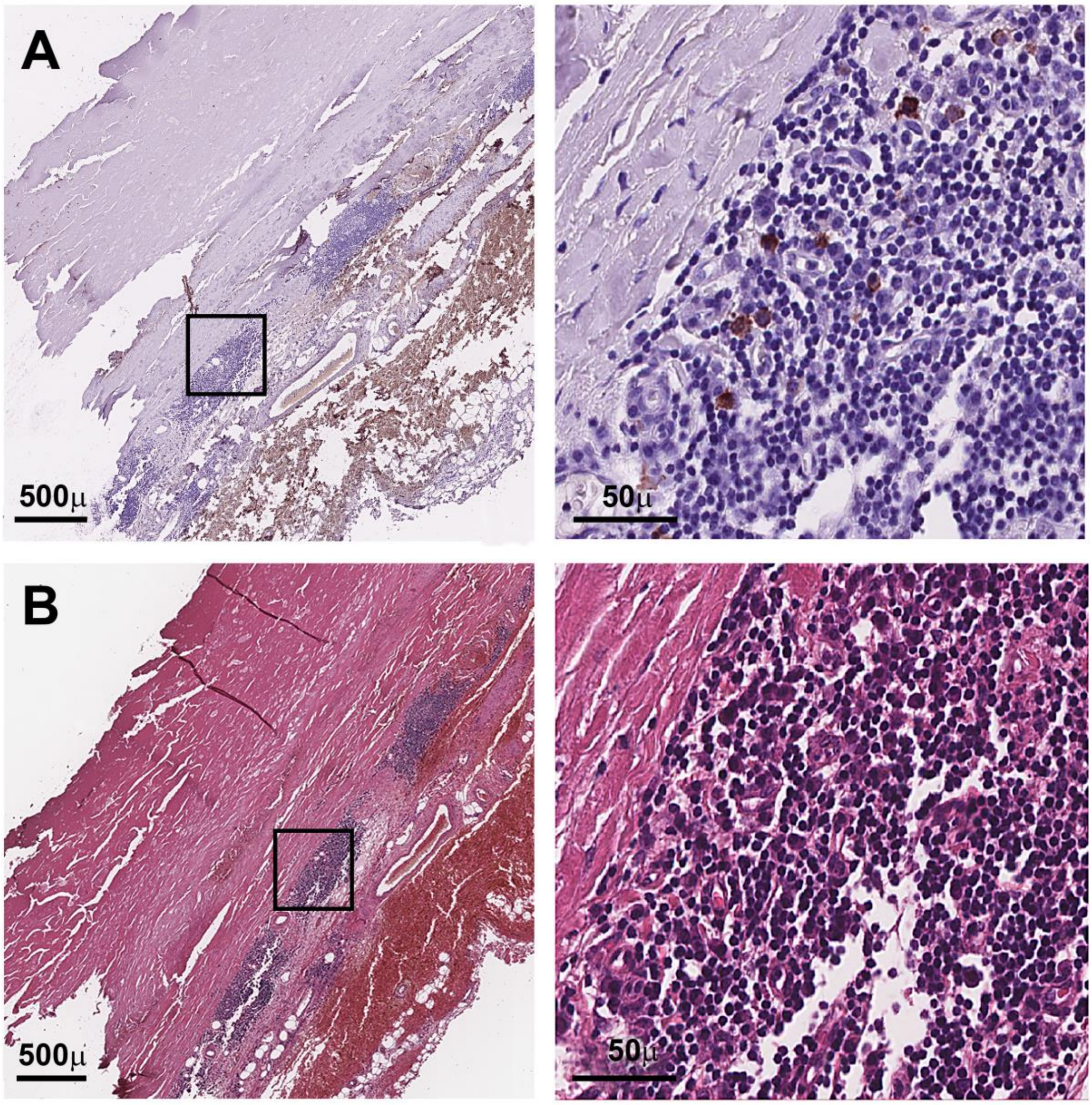

Suppl. Figure S1: Additional example of IgG4 positive AAA staining: (A) Overview and close up of IgG4-immunostaining from additional patients with positivity score 1. **(B)** Corresponding overview and close up of HE staining. (scale bar 200µm, 50µm respectively, all photos lumen oriented upwards).

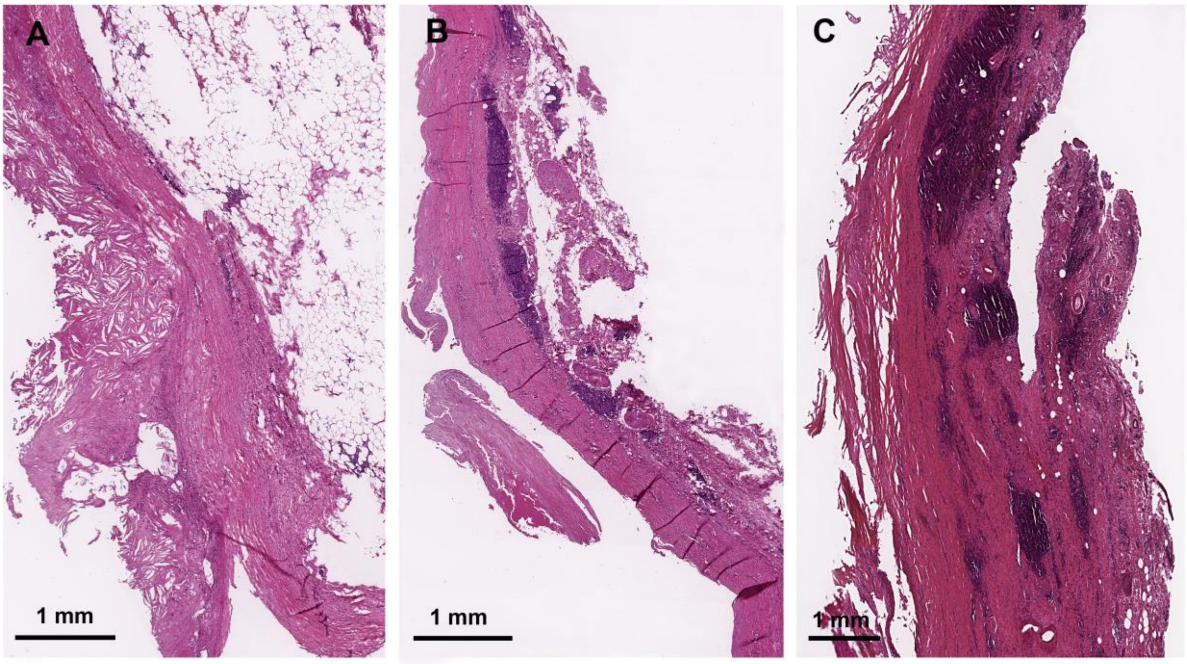

Suppl. Figure S2: Degree of inflammation in AAA vessel wall. (A) No to very low level of infiltrate. **(B)** Intermediate level of infiltrate. **(C)** High levels of inflammatory infiltrate (scale bar 1mm; lumen oriented sidewards; HE staining).

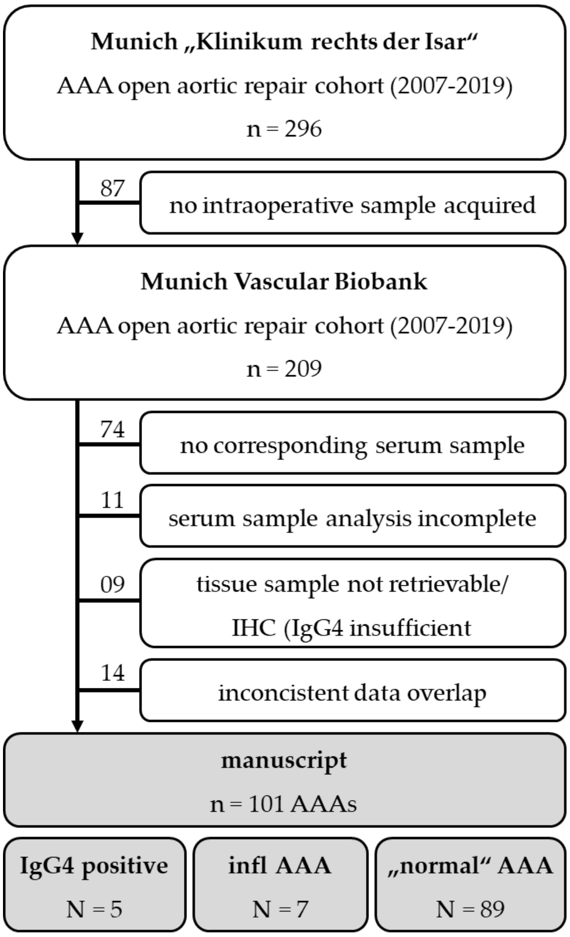

Suppl. Figure S3: Inclusion flow chart.

		patient cohort	IgG4 positive AAA	inflammatory AAA
		N=101	N=5	N=7
**AAA characteristics**				
growt[illegible]	slow	10 (9.9)	2 (40)	1 (14.3)
	intermediate	22 (21.8)	0	3 (42.9
	fast	13 (12.9)	0	0
**procedural details and clinical outcome**				
surgical complication		20 (19.8)	1 (20)	2 (28.6)
SSI		12 (11.9)	1 (20)	1 (14.3)
leg ischemia		7 (6.9)	0	1 (14.3)
bleeding		5 (5)	0	0
bowel ischemia		1 (1)	0	0
medical complication		40 (39.6)	2 (40)	1 (14.3)
urinary tract infection		7 (6.9)	1 (20)	0
acute kidney failure		11 (10.9)	0	0
ischemic colitis		5 (5)	0	0
myocardial infarction		3 (3)	0	0
stroke		1 (1)	0	0
lung edema		7 (6.9)	1 (20)	0
pneumonia		11 (10.9)	0	1 (14.3)
other		2 (2)	0	0

Suppl. Table S1: Additional AAA characteristics and short-term clinical outcomes. SSI = surgical site infection. Continuous variables are shown as mean ± one standard deviation (SD). Categorical variables are shown as absolute number and percentage.
